# Eplerenone reduces lymphangiogenesis in the contralateral kidneys of UUO rats

**DOI:** 10.1038/s41598-024-60636-z

**Published:** 2024-05-01

**Authors:** Juan Hao, Panpan Qiang, Lili Fan, Yunzhao Xiong, Yi Chang, Fan Yang, Xiangting Wang, Tatsuo Shimosawa, Shengyu Mu, Qingyou Xu

**Affiliations:** 1 Graduate School, Hebei University of Chinese Medicine, Shijiazhuang, China; 2 Hebei Key Laboratory of Integrative Medicine on Liver-Kidney Patterns, Hebei University of Chinese Medicine, Shijiazhuang, China; 3 Institute of Integrative Medicine, College of Integrative Medicine, Hebei University of Chinese Medicine, Shijiazhuang, China; 4 Department of Clinical Laboratory, School of Medicine, International University of Health and Welfare, Narita, Chiba Japan; 5 Department of Pharmacology and Toxicology, University of Arkansas for Medical Sciences, Little Rock, AR USA; 6 Shijiazhuang Hospital of Traditional Chinese Medicine, Shijiazhuang, China

**Keywords:** Renal fibrosis, Lymphangiogenesis

## Abstract

Inflammation and fibrosis often occur in the kidney after acute injury, resulting in chronic kidney disease and consequent renal failure. Recent studies have indicated that lymphangiogenesis can drive renal inflammation and fibrosis in injured kidneys. However, whether and how this pathogenesis affects the contralateral kidney remain largely unknown. In our study, we uncovered a mechanism by which the contralateral kidney responded to injury. We found that the activation of mineralocorticoid receptors and the increase in vascular endothelial growth factor C in the contralateral kidney after unilateral ureteral obstruction could promote lymphangiogenesis. Furthermore, mineralocorticoid receptor activation in lymphatic endothelial cells resulted in the secretion of myofibroblast markers, thereby contributing to renal fibrosis. We observed that this process could be attenuated by administering the mineralocorticoid receptor blocker eplerenone, which, prevented the development of fibrotic injury in the contralateral kidneys of rats with unilateral ureteral obstruction. These findings offer valuable insights into the intricate mechanisms underlying kidney injury and may have implications for the development of therapeutic strategies to mitigate renal fibrosis in the context of kidney disease.

## Introduction

Chronic kidney disease (CKD) affects more than 10% of the global population^1–3^, and a significant number of these CKD cases result from acute kidney injury. Renal fibrosis is a common pathological characteristic of CKD that is frequently observed in cases of postrenal obstructive nephropathy^4^. The unilateral ureteral obstruction (UUO) model is a well-established model for studying obstructive nephropathy and renal fibrogenesis. Over the past few decades, numerous studies using this model have identified several molecular mechanisms responsible for injury and fibrosis in obstructed kidneys. However, investigations of the pathological mechanisms leading to chronic renal injury and fibrosis in the contralateral kidney, which significantly influence renal function and long-term outcomes, have been limited. Understanding these mechanisms is crucial for gaining further insights into the development and progression of CKD and for devising effective therapeutic strategies to manage this prevalent and debilitating condition.

In our previous studies, we showed that elevated aldosterone levels could stimulate mineralocorticoid receptor (MR) activation in the contralateral kidneys of rats with long-term UUO 180 days after UUO surgery^5–7^. MR activation, promoted macrophage-to-myofibroblast transition (MMT), which significantly contributed to renal fibrogenesis^5–7^. However, the pathogenesis of renal fibrosis is multifaceted and involves various factors, such as inflammatory stimulation, MMT, and lymphangiogenesis^4,7–10^. The involvement of MR activation in other pathogenic mechanisms leading to renal fibrosis has yet to be determined. Previous studies have suggested a role for aldosterone-induced MR activation in angiogenesis and vascular remodeling^11,12^. These observations have sparked our curiosity regarding whether MR activation plays a role in lymphatic endothelial cell (LEC) proliferation and secretion, thereby contributing to renal inflammation and fibrosis in the contralateral kidneys of rats with long-term UUO. Investigating this possibility could provide valuable insights into the mechanisms involved in the chronic development of renal fibrosis.

Lymphangiogenesis is primarily stimulated by increases in vascular endothelial growth factor C (VEGFC) and its specific receptor vascular endothelial growth factor receptor-3 (VEGFR3)^13–15^, and a significant source of VEGFC production is monocytes/macrophages^8,16,17^. Our previous study revealed increased infiltration of macrophages into the contralateral kidneys of rats with UUO^7^. Therefore, it is reasonable to hypothesize that the contralateral kidneys of rats with UUO exhibit higher VEGFC production than kidneys from control animals, which may result in increased lymphangiogenesis. However, an intriguing question that remains to be answered is whether MR activation can also stimulate VEGFC production in macrophages. This question is a critical aspect of our ongoing study, since understanding the role of MR activation in modulating VEGFC production by macrophages could shed light on the complex mechanisms driving lymphangiogenesis in the context of renal fibrosis.

Because the MR inhibitor eplerenone attenuates renal fibrosis in the contralateral kidneys^5,7^, we hypothesize that MR activation contributes to the pathogenesis of renal lymphangiogenesis and collagen secretion in the contralateral kidneys of animals with long-term UUO. Therefore, in this study, we aimed to examine the role of MR in stimulating lymphangiogenesis in the contralateral kidneys of rats with UUO (UUO-CLK group). Our objective was to provide novel insights into the mechanisms underlying chronic renal fibrosis in the contralateral kidneys following UUO injury. By elucidating these mechanisms, we hope to improve our understanding of renal fibrosis development and identify new therapeutic targets to mitigate its progression.

## Results

### Eplerenone effectively mitigated contralateral renal injury and fibrosis induced by prolonged UUO

**Figure 1:**
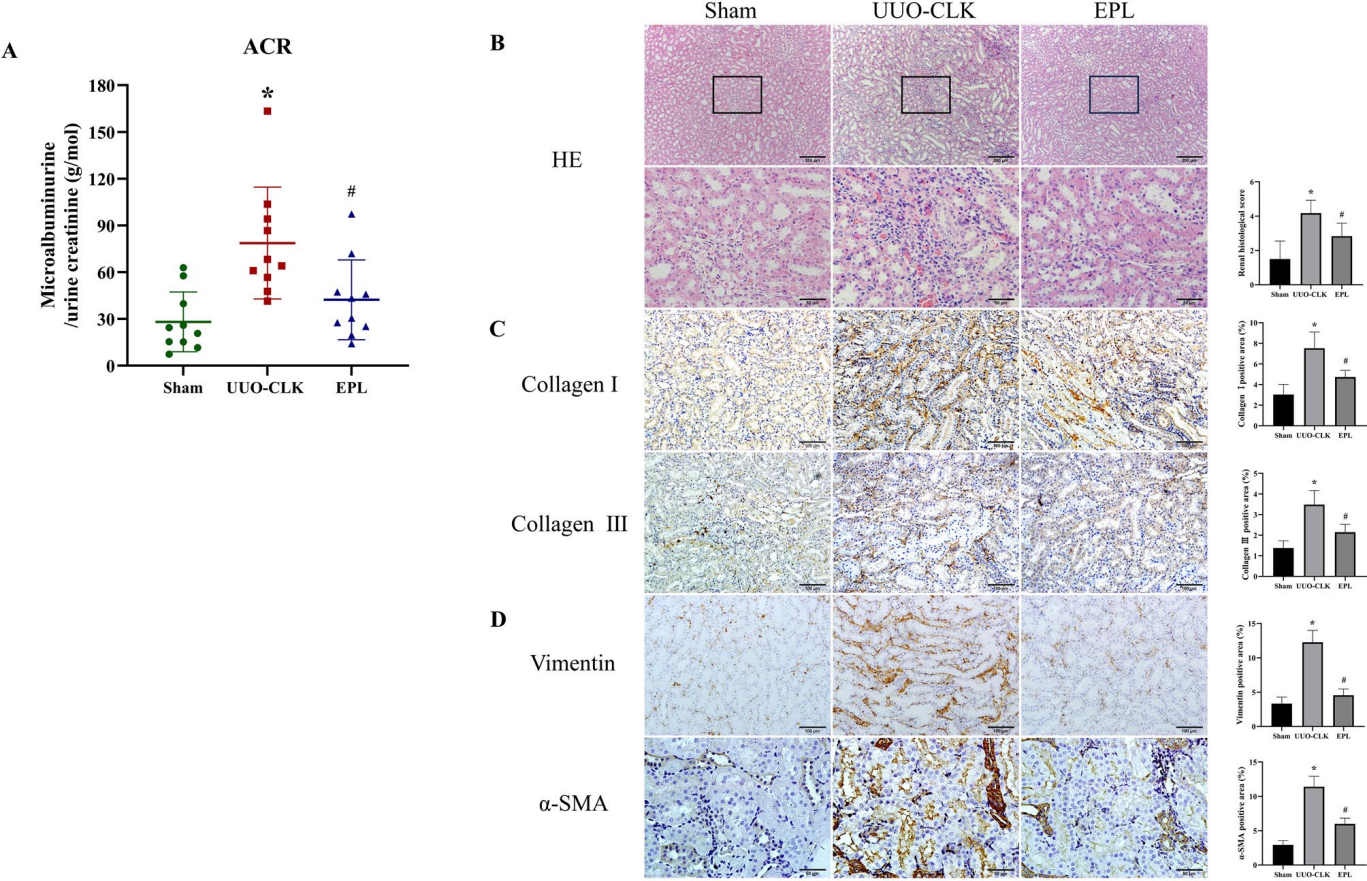
Eplerenone mitigated contralateral renal injury and fibrosis induced by long-term UUO. **(A)** The ACR was evaluated to determine renal function (*n* = 10). **(B)** HE staining was used to assess changes in kidney morphology, including tubular dilation, arrangement, and inflammatory cell infiltration (scale bars = 50 μm, 200 μm). Immunohistochemistry was performed to analyze the expression of the fibrosis-related proteins collagen I, collagen III **(C)**, vimentin, and α-SMA **(D)** in kidney tissue (scale bars = 50/100 μm). The data are presented as the mean ± SD (*n* = 6); ^*^P < 0.05 vs. Sham; ^#^*P* < 0.05 vs. UUO-CLK.

UUO is a classic model for studying renal inflammation and fibrosis. In addition to causing acute injury in the obstructed kidney, the contralateral kidney also undergoes fibrosis after long-term UUO (180 days), which adversely affects renal function over time. We tested renal function in the rats, and compared with those in the sham group, the blood urea nitrogen (BUN) and serum creatinine (Scr) levels were significantly altered, indicating that kidney function was impaired^7^. The microalbuminuria-to-creatinine ratio (ACR) showed the same change (Fig.1A). In the present study, we used hematoxylin and eosin (HE) staining to examine morphological and pathological alterations in the contralateral kidneys of rats 180 days after UUO surgery (Fig.1B). In comparison to those in the sham group, there was significant dilation of renal tubules, disordered arrangement, and inflammatory cell infiltration in the interstitium in the UUO-CLK group. Notably, these pathological changes were mitigated by eplerenone treatment (Fig.1B). To confirm the inhibitory effect of eplerenone on fibrosis in the contralateral kidneys of rats with UUO, we examined the protein expression of the fibrogenic markers collagen I and collagen III by immunohistochemistry (Fig.1C). The results revealed substantial upregulation of collagen I and collagen III expression in the UUO-CLK group compared to the sham group (*P*< 0.05). Importantly, eplerenone treatment significantly downregulated the expression of these fibrogenic markers (*P*< 0.05) (Fig.1C). Furthermore, α-SMA and vimentin, which are indicative of fibrotic processes, were examined by immunohistochemical staining (Fig.1D). In the UUO-CLK group, there was a marked increase in the expression of α-SMA and vimentin compared to that in the Sham group (*P*< 0.05). In contrast, treatment with eplerenone led to a substantial reduction in the expression of α-smooth muscle actin (α-SMA) and vimentin (*P*< 0.05). In addition to changes in fibrosis, inflammation in the kidney is an important indicator of injury. Western blot analysis revealed that the expression of tumor necrosis factor-α (TNF-α) and interleukin-1β (IL-1β) was upregulated in the UUO-CLK group, suggesting that UUO induced inflammation, and eplerenone reduced the degree of inflammation (Supplementary Fig.1). These findings suggest that eplerenone effectively counteracts renal injury and fibrosis in the contralateral kidney after long-term UUO.

### Eplerenone effectively attenuated lymphangiogenesis induced by long-term UUO in the contralateral kidney

**Figure 2:**
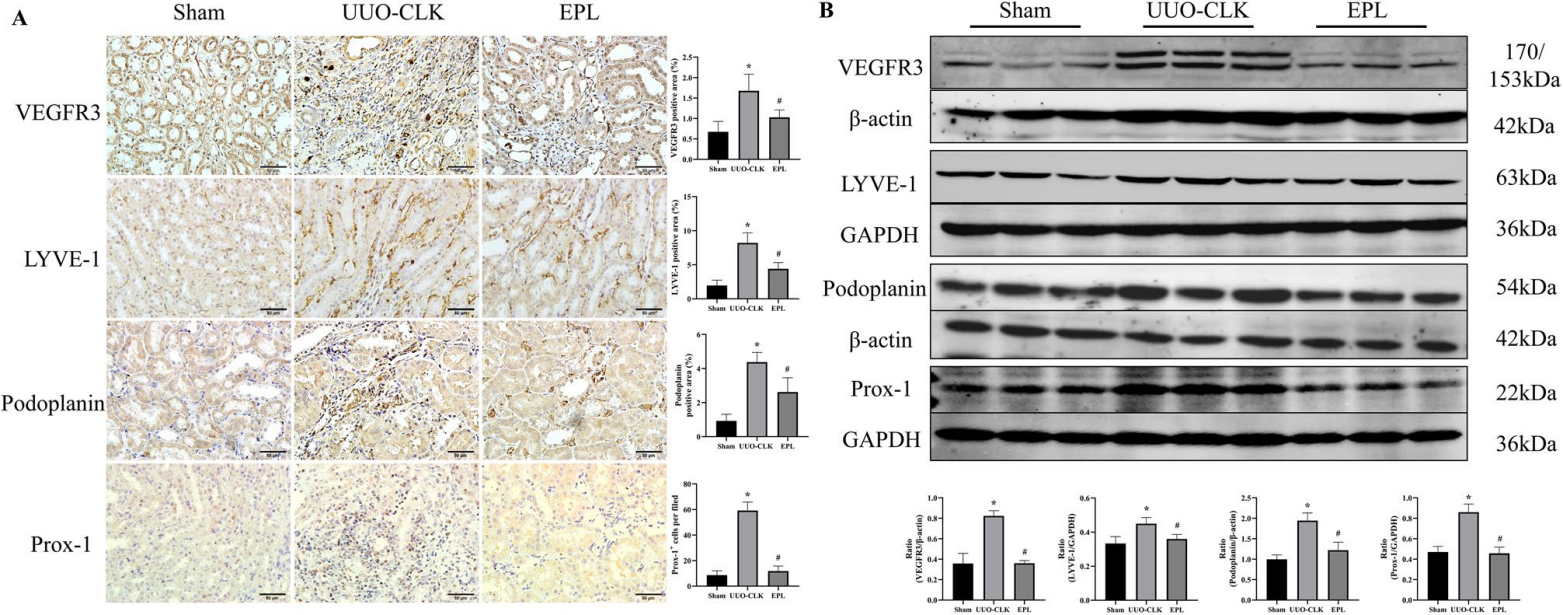
Eplerenone attenuated lymphangiogenesis induced by long-term UUO in the contralateral kidney. **(A)** Immunohistochemical staining using antibodies against VEGFR3, LYVE-1, podoplanin, and Prox-1 to examine lymphangiogenesis in the contralateral kidneys of rats with UUO (scale bars = 50 μm, *n* = 6). **(B)** The same antibodies were used for Western blot analysis to confirm the quantification of the imaging data (*n* = 3). The data are presented as the mean ± SD; ^*^P < 0.05 vs. Sham; ^#^*P* < 0.05 vs. UUO-CLK.

Recent studies have highlighted a connection between the development of fibrosis in CKD (or the chronic phase of acute kidney injury) and the expansion of lymphatic vessels in the kidney. To investigate whether this phenomenon is also observed in the contralateral kidneys of animals subjected to long-term UUO, we performed immunostaining (Fig.2A) and Western blot analysis (Fig.2B) using lymphatic-specific markers, including VEGFR3, lymphatic vessel endothelial receptor-1 (LYVE-1), podoplanin, and Prox1. In the contralateral kidneys of rats subjected to long-term UUO, we observed a significant increase in lymphangiogenesis, as evidenced by the increased staining of the lymphatic-specific markers VEGFR3, LYVE-1, podoplanin, and Prox-1 compared to that in the kidneys in the Sham group. Western blot analysis using the same antibodies verified these imaging findings (Fig.2B). Interestingly, the induction of lymphangiogenesis in the contralateral kidney by long-term UUO was mitigated by eplerenone treatment. This finding suggested a positive correlation between renal lymphangiogenesis and fibrosis, which was consistent with previous research findings, and the activation of MR may be involved in this correlation.

### MR activation increased macrophage infiltration and VEGFC production in the contralateral kidneys of rats with long-term UUO

**Figure 3:**
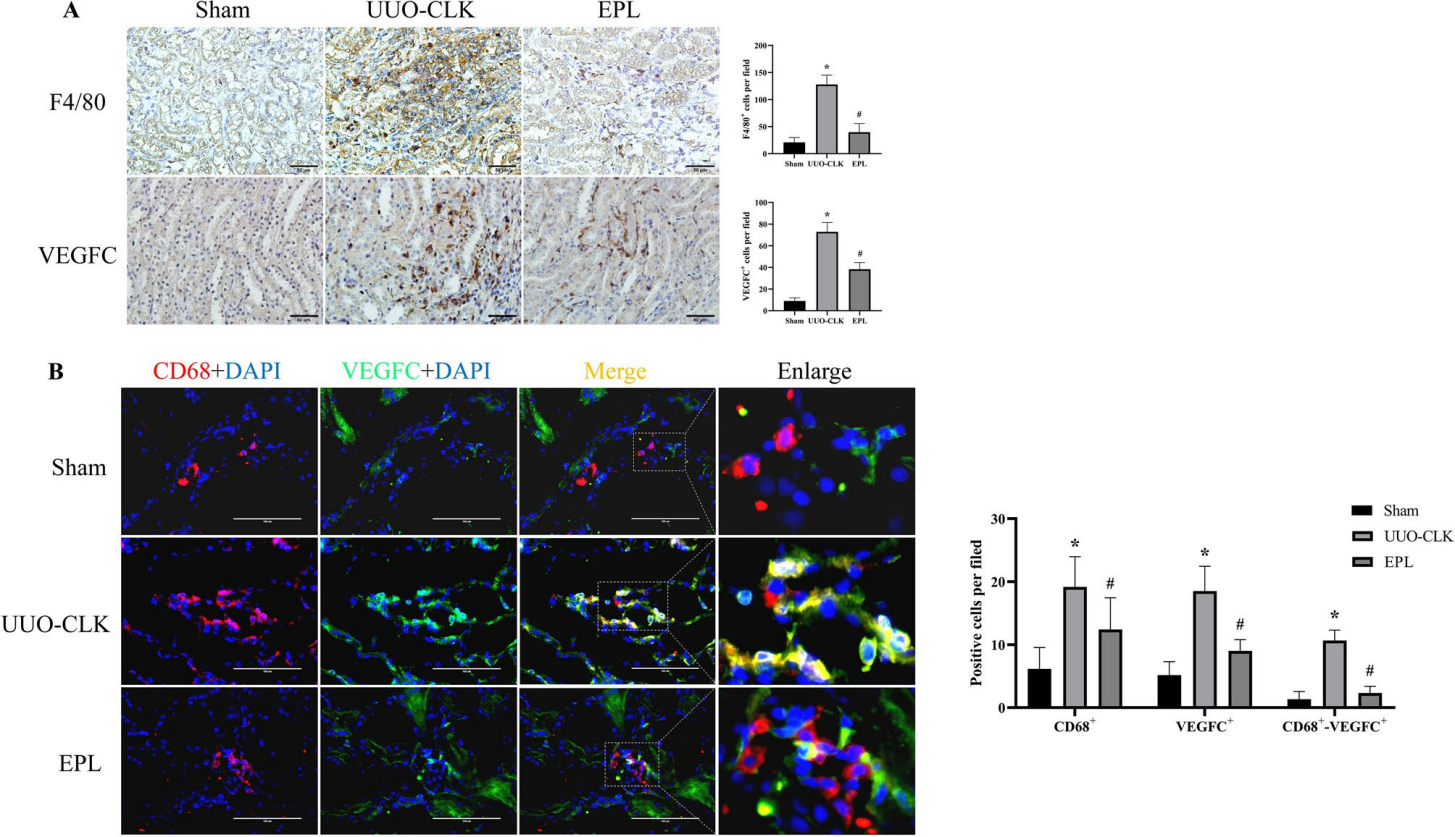
Eplerenone inhibited macrophage infiltration and VEGFC production in the contralateral kidneys of rats with UUO. **(A)** Immunohistochemical staining to examine the protein expression of VEGFC and the macrophage marker F4/80 (scale bars = 50 μm, *n* = 6). **(B)** To verify that VEGFC was mainly secreted by macrophages, we used triple immunofluorescence staining to examine the macrophage marker CD68 (TRITC, red), VEGFC (FITC, green) and the nucleus (DAPI, blue). VEGFC^+^ and CD68^+^ areas represent macrophages that secrete VEGFC (scale bar = 100 μm, *n* = 6). The data are presented as the mean ± SD; ^*^P < 0.05 vs. Sham; ^#^*P* < 0.05 vs. UUO-CLK.

VEGFC is a potent factor that stimulates lymphangiogenesis through its receptor VEGFR3. A primary source of VEGFC production is macrophages. In the contralateral kidneys of rats with UUO, we observed increased levels of macrophage infiltration (F4/80^+^cells) and VEGFC production compared to those in Sham rats (Fig.3A). This finding suggested that the increased infiltration of macrophages into the contralateral kidneys of rats with UUO increased VEGFC production within the renal interstitial space, ultimately leading to increased lymphangiogenesis. As expected, renal macrophage infiltration and VEGFC expression were suppressed by eplerenone treatment (Fig.3A). Merged images showing co-staining of macrophages and VEGFC in the kidneys revealed increased macrophage infiltration in the contralateral kidneys of rats with long-term UUO and increased production of VEGFC by these macrophages compared to those in Sham kidneys. Furthermore, this increase in VEGFC production was inhibited by eplerenone (Fig.3B). To better explain the relationship between VEGFC and macrophages, RAW264.7 cells were cultured in vitro and then treated with aldosterone. Western blot analysis showed that VEGFC secretion by macrophages was increased (Supplementary Fig.2). Moreover, we found that aldosterone activated MR in RAW264.7 cells, but there was no significant difference in the total expression of MR; aldosterone increased nuclear MR expression, and eplerenone blocked the nuclear translocation of MR (Supplementary Fig.3). These findings suggest that MR activation can stimulate macrophages to produce more VEGFC in the contralateral kidneys of rats subjected to long-term UUO.

### MR activation directly stimulated proliferation and tube formation in LECs.

**Figure 4:**
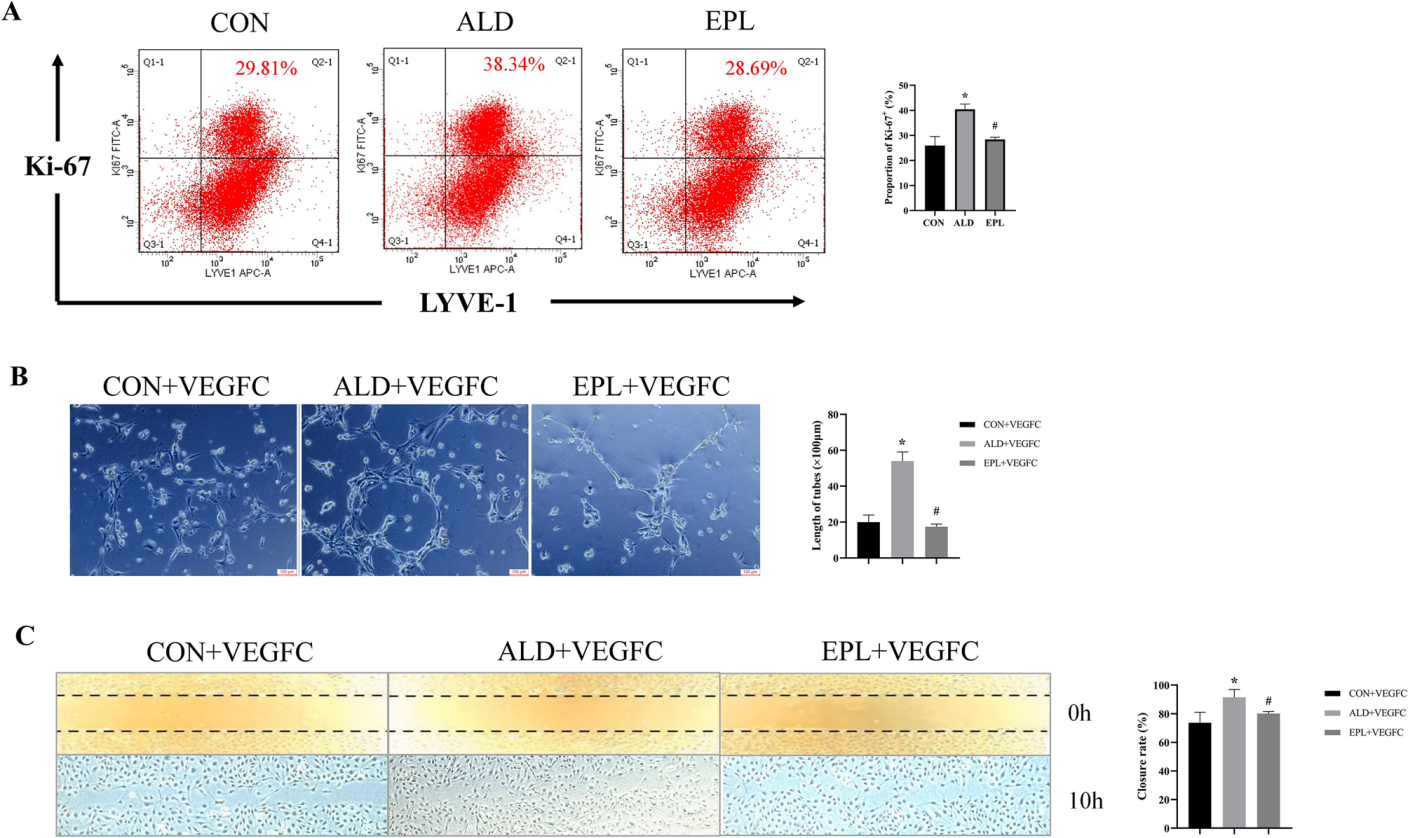
MR activation directly stimulated proliferation and tube formation in LECs. **(A)** hLEC proliferation was measured by two-color flow cytometry. The numbers in the upper right corner indicate the percentage of proliferating cells labeled Ki-67^+^. **(B)** Cells were grown in conditioned media and placed in the three-dimensional matrix of an ECM gel. Then, capillary tube formation was photographed after 10 h (scale bars = 100 μm). **(C)** hLECs were then scratched and stimulated with 100 ng/ml VEGFC in the CON, ALD and EPL groups. The images shown were obtained at 0 h (immediately after wounding) and 10 h after wounding. The data are presented as the means ± SD (*n* = 3); *P < 0.05 vs. CON; ^#^*P* < 0.05 vs. ALD.

Previous results have shown the impact of MR activation on macrophages and their role in stimulating renal lymphangiogenesis, and it is reasonable to consider whether MR activation directly influences the proliferation of lymphatic vessels. Thus, we performed in vitro experiments in which aldosterone was administered to cultured human LECs (hLECs). We examined cell proliferation using flow cytometry and the cell proliferation marker Ki67. Our findings revealed that aldosterone significantly increased the proportion of hLECs with high expression of Ki67, indicating an increase in cell proliferation. Notably, this effect was blocked when hLECs were preincubated with the MR blocker eplerenone (Fig.4A). Additionally, we examined the behavior of hLECs cultured with VEGFC. The wound healing experiments showed that aldosterone increased cell migration, and in cultured hLECs, it promoted tube formation (Fig.4B,C). Notably, preincubation with the MR blocker eplerenone attenuated the additional effects of aldosterone but did not affect VEGFC-induced cell growth in hLECs (Fig.4).

### Eplerenone mitigated the lymphatic endothelial-to-myofibroblast transition (EndMT) induced by MR stimulation

**Figure 5:**
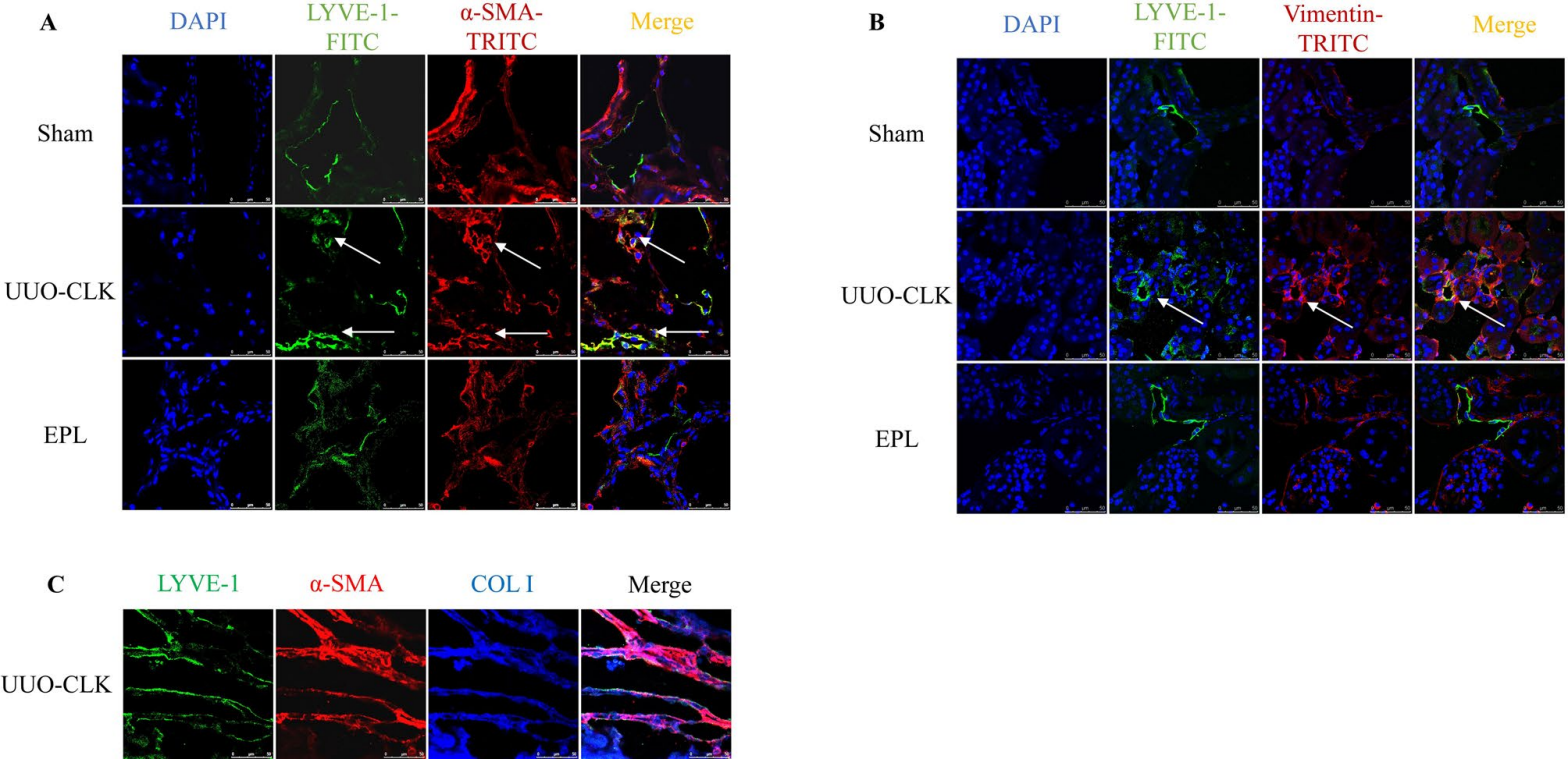
Eplerenone alleviated renal fibrosis by reducing EndMT. To verify whether lymphatic EndMT occurred, we used triple immunofluorescence staining to examine the lymphatic marker LYVE-1 (FITC, green), the myofibroblast markers α-SMA **(A)** and vimentin **(B)** (TRITC, red) and the nucleus (DAPI, blue). LYVE-1^+^ and α-SMA^+^/Vimentin^+^ areas represent EndMT (scale bar = 50 μm). **(C)** Immunofluorescence staining of kidney sections with antibodies against LYVE-1 (FITC, green), α-SMA (TRITC, red), and collagen I (DyLight 405, blue) to determine the involvement of EndMT in renal fibrosis (scale bar = 50 μm).

While EndMT has been extensively studied in the context of myofibroblast formation, most research has focused on blood vessel endothelial cells. However, recent findings have shown that EndMT can also occur in LECs, thereby contributing to tissue fibrosis in systemic sclerosis. We hypothesized that lymphatic EndMT might also play a role in the contralateral kidney in rats with long-term UUO and promote renal fibrosis. Compared to Sham kidneys, the contralateral kidneys of rats with long-term UUO exhibited increased co-expression of the specific lymphatic marker LYVE-1 with the activated myofibroblast markers α-SMA (Fig.5A) or vimentin (Fig.5B). In addition, we found that LECs transformed into myofibroblasts and secreted collagen components, as shown by fluorescent triple-stain labeling of LECs, myofibroblasts and collagen I, which provided evidence for the involvement of EndMT in renal fibrosis (Fig.5C). To further determine the source of collagen, we used confocal microscopy, Z-stack series images and dynamic observations to examine collagen secretion in the contralateral kidney in rats with long-term UUO (Supplementary Fig.4). Intriguingly, treatment with eplerenone, which is a potent MR blocker, attenuated these effects. This raises the question of whether eplerenone-mediated suppression of lymphangiogenesis in the contralateral kidney, as shown in Fig.2, is the sole reason for this attenuation or whether MR inhibition can directly block lymphatic EndMT.

**Figure 6:**
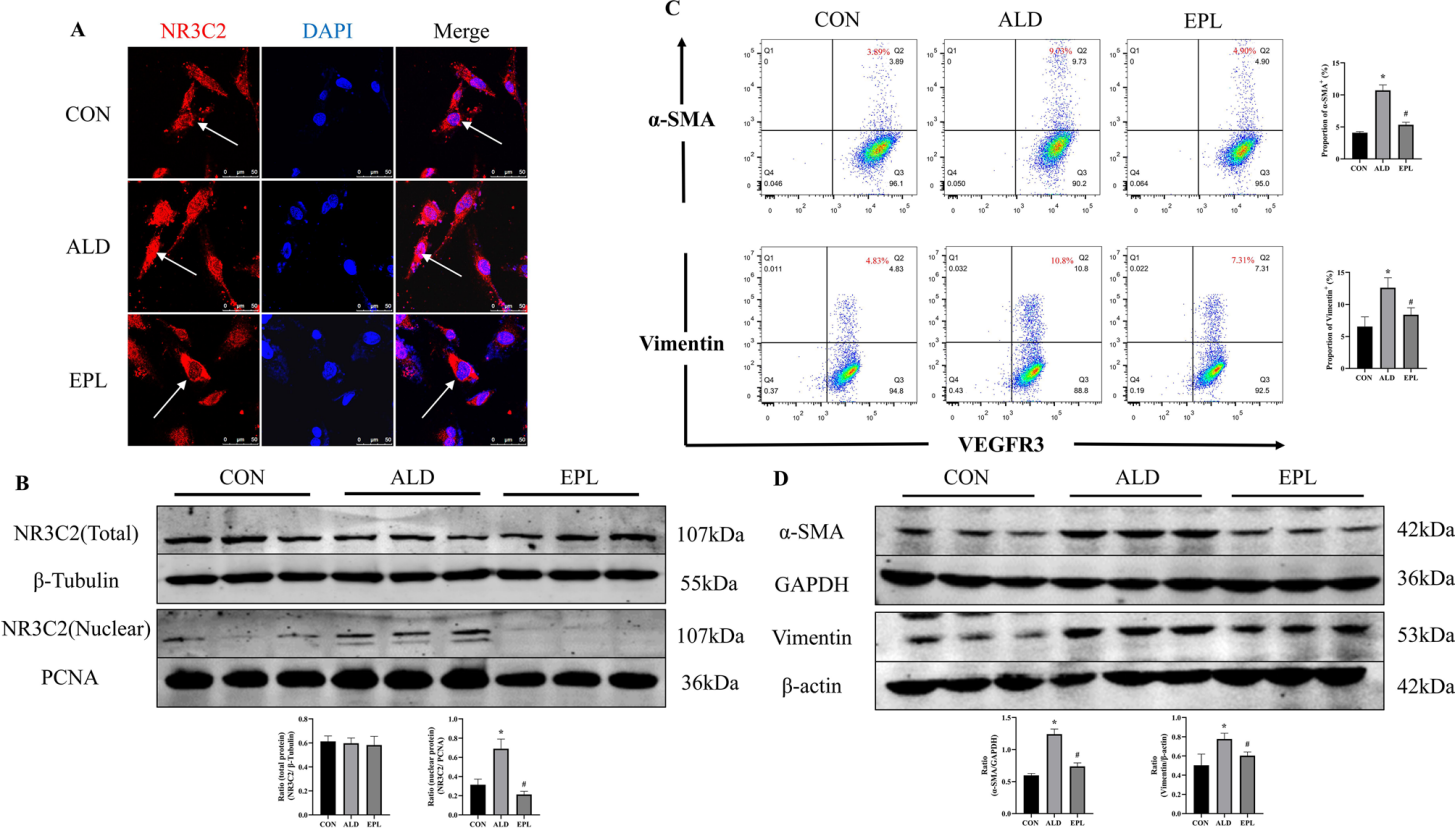
Eplerenone mitigated EndMT induced by MR stimulation. **(A)** Immunocytofluorescence analysis of hLECs with an antibody against the MR marker NR3C2 (TRITC, red) to determine the location of MR expression, and nuclei were stained with DAPI (blue) (scale bars = 50 μm). **(B)** Western blot analysis of MR in total and nuclear protein fractions of hLECs. **(C)** Flow cytometric analysis of the coexpression of the myofibroblast marker α-SMA or vimentin and the lymphatic marker VEGFR3 in hLECs. Q2 shows the percentage of LECs undergoing the myofibroblast transition. **(D)** Western blot analysis of the expression of α-SMA and vimentin in hLECs. hLECs were treated with aldosterone (10^−7^mol/L) and pretreated with eplerenone (10^−6^mol/L) 1 h prior to aldosterone treatment. The data are presented as the means ± SD (*n*= 3);^*^P< 0.05 vs. CON;^#^*P*< 0.05 vs. ALD.

To investigate whether MR activation directly participates in the lymphatic EndMT process, we administered aldosterone with or without eplerenone to cultured hLECs. As expected, aldosterone induced the nuclear translocation of MR in hLECs, as shown by immunofluorescence analysis and Western blotting (Fig.6A,B), and this effect has been commonly observed in most mammalian cells. Furthermore, flow cytometry and Western blot analysis showed that aldosterone increased the expression of the myofibroblast markers α-SMA and vimentin on the surface and in the total lysates of hLECs (Fig.6C,D). Importantly, the upregulation of these factors was impaired by the MR blocker eplerenone. These results suggest that MR activation plays a crucial role in promoting lymphatic EndMT, thereby contributing to fibrosis in the contralateral kidneys of rats with long-term UUO.

## Discussion

Renal inflammation and fibrosis are common during CKD progression and can lead to renal dysfunction and kidney injury. The pathogenesis of renal inflammation and fibrosis is closely related to interstitial lymphangiogenesis, and inhibiting lymphangiogenesis ameliorates renal inflammation and fibrogenesis^4^. Our previous study showed that after long-term UUO (180 days), the contralateral kidney exhibited inflammation and fibrosis^5,7^. As shown in Fig.1and Supplementary Fig.1, the contralateral kidneys of rats with UUO exhibited robust inflammatory cell infiltration and fibrotic changes, and renal function was significantly impaired. This effect may be related to the inflammatory response induced by aldosterone, and treatment with eplerenone could reduce damage to the contralateral kidneys in rats with UUO. In addition, we also observed lymphangiogenesis in areas with obvious inflammatory cell infiltration in the interstitium of the contralateral kidney. Whether this renal pathogenesis is associated with interstitial lymphangiogenesis and the molecular mechanisms mediate renal lymphangiogenesis in the contralateral kidney are two important questions that, if answered, will not only add to our knowledge of the pathogenic progression of CKD but also provide critical insights into protecting the contralateral kidney after obstructive nephropathy, thereby improving renal function.

The main physiological function of the lymphatic system is to maintain the tissue-fluid balance. The lymphatic network remains relatively stationary after initial development and maturation but regrows and remodels when stimulated. New lymphangiogenesis often occurs at sites of tissue damage, interstitial fluid overload, hyperglycemia, and inflammation^18^. Lymphangiogenesis helps to clear inflammatory cells from the damaged tissue environment, thereby alleviating inflammation and preventing fibrosis. However, this study revealed that lymphangiogenesis exacerbated UUO-induced contralateral intrarenal inflammation and subsequent fibrosis, and eplerenone inhibited lymphangiogenesis and alleviated renal injury. These findings are consistent with those studies of lymphangiogenesis in other CKDs, such as lupus nephritis, antineutrophil cytoplasmic antibody-associated nephritis, tubulointerstitial nephritis, focal segmental glomerulosclerosis, crescentic nephritis, type II diabetic nephropathy, and IgA nephropathy, which exhibit significantly increased lymphangiogenesis compared with controls^4,19,20^. During chronic inflammation, lymphangiogenesis is a kind of disorderly expansion that can hinder the clearance of immune cells, leading to the accumulation of macrophages, dendritic cells, T and B lymphocytes, and fibroblasts, thereby exacerbating the chronic inflammatory response and ultimately leading to fibrosis^21^. Thus, the extent to which lymphangiogenesis is protective rather than maladaptive may depend on the environment or the stage of disease progression. Lymphangiogenesis is strongly associated with renal injury and interstitial fibrosis during chronic inflammation. Therefore, treatments that target lymphangiogenesis can restore clearance, modulate the inflammatory environment, and prevent fibrotic remodeling during CKD.

During renal fibrosis, macrophages can promote lymphangiogenesis in two ways: transdifferentiation into lymphatic vessels and the secretion of lymphangiogenic factors such as VEGFC, VEGFD and VEGFA in response to inflammatory mediators^22^. VEGFC is central to lymphangiogenesis in kidney disease and triggers lymphangiogenesis primarily through the activation of VEGFR3. VEGFC highly expressed in macrophages in rat residual kidney tissue, mice with UUO, human IgA nephropathy biopsy samples, diabetic kidney disease (DKD), and chronic allograft rejection^8,21^. We confirmed the association between the increase in VEGFC and macrophages by costaining for a macrophage marker (CD68) and VEGFC. This finding was also validated in RAW264.7 cells cultured in vitro and suggested that UUO-induced macrophage infiltration generated a favorable microenvironment for lymphangiogenesis and was involved in lymphangiogenesis through the secretion of VEGFC. These macrophages, which may originate from the bone marrow or from in situ proliferation of kidney macrophages, may be activated by MR, the inflammatory cytokines IL-1β and TNF-α or hypoxia-inducible factor^21,23–26^. In addition, renal tubular epithelial cells can also be sources of VEGFC in response to renal injury^24,27^, which remains to be confirmed in future studies.

Lymphangiogenesis is related to chronic inflammation, and the occurrence of chronic inflammation and macrophage infiltration is associated with increased aldosterone levels. The present study focused on the impact of MR activation and eplerenone treatment on renal injury, fibrosis, and lymphangiogenesis, which is mediated by lymphatic EndMT. First, our research demonstrated that MR activation induced an inflammatory response; and HE staining revealed inflammatory cell infiltration in the contralateral kidney, and Western blot analysis revealed significantly increased expression of the inflammatory factors TNF-α and IL-1β, while eplerenone effectively mitigated contralateral renal injury and fibrosis induced by long-term UUO. As shown in Fig.1, the contralateral kidney, which is often overlooked in UUO studies, was revealed to be a site that was vulnerable to fibrosis, which could adversely affect renal function. The ability of eplerenone to ameliorate these pathological changes in the contralateral kidney underscores its potential therapeutic value in CKD associated with fibrosis. Furthermore, we extended the understanding of the role of MR activation in the regulation of lymphangiogenesis. As shown in Fig.4, the costaining of Ki-67 with LYVE-1 and wound healing and tube formation assays confirmed the role of aldosterone in promoting the proliferation, migration, and tube formation of LECs. Our study provides compelling evidence that MR activation enhances LEC proliferation and tube formation, directly linking MR activation to the promotion of lymphangiogenesis. These findings may have significant implications for understanding the mechanisms underlying fibrosis and lymphatic expansion in renal diseases.

How is lymphangiogenesis involved in renal fibrosis? Our investigation examined lymphatic EndMT, which is a process that recently gained attention for its contribution to fibrosis in various tissues^28–30^. Repetitive or severe damage in chronic inflammatory diseases results in an abnormal tissue repair response characterized by the excessive accumulation of collagen and fibronectin. Organ fibrosis may be closely related to lymphatic system abnormalities^31^. Co-staining of an LECs marker (LYVE-1) and myofibroblast markers (α-SMA or vimentin), showed that some cells expressed both markers, which may indicate that some LECs are involved in the transformation into myofibroblasts. The triple staining results for LYVE-1, α-SMA and collagen I showed that these transformed cells could participate in renal fibrosis through the production of collagen. We provide evidence suggesting that MR activation plays a pivotal role in promoting lymphatic EndMT. This finding suggested that inhibiting MR with drugs such as eplerenone could inhibit this transition and reduce fibrosis. In summary, our study shows the multifaceted effects of MR activation on renal injury and fibrosis. We showed that eplerenone could mitigate renal damage, lymphangiogenesis, and lymphatic EndMT in the context of long-term UUO, suggesting potential therapeutic options for CKD associated with fibrosis and lymphatic dysfunction.

While our study provides valuable insights, it is important to acknowledge certain limitations that may affect the generalizability and interpretability of our findings. First, our in vitro experiments focused on cultured hLECs. These cells are a valuable platform for studying cellular responses to MR activation, but they may not fully represent the in vivo conditions within the kidney. Further studies using animal models and human tissues are necessary to corroborate our in vitro findings. Second, our study primarily investigated the effects of eplerenone as an MR blocker. While eplerenone is a well-established MR antagonist^32^, future investigations could explore the use of other MR antagonists or alternative therapeutic strategies to confirm and expand upon our findings. Third, our research highlights the role of MR activation in promoting lymphatic EndMT, but the specific molecular mechanisms underlying this process have yet to be fully elucidated. Future studies should examine these mechanisms to uncover new therapeutic targets for fibrotic diseases.

Building upon our current findings, several avenues for future research have emerged. One future direction involves molecular mechanisms. Elucidating the molecular pathways involved in MR-mediated lymphatic EndMT will be crucial. Investigating how MR activation interacts with signaling pathways and transcription factors could provide a deeper understanding of this process and potential targets for intervention. In this study, MR activation was thought to play a role; therefore, eplerenone, which is an MR blocker, was used to inhibit MR activation, and direct aldosterone stimulation will be necessary to verify these findings in subsequent studies. Another important future study is to examine lymphatic function. Investigating the functional consequences of lymphatic changes in renal diseases is crucial. Future studies should examine how alterations in lymphatic function impact the progression of renal fibrosis and overall kidney health.

In conclusion, our study reveals the multifaceted effects of MR activation on renal injury, fibrosis, lymphangiogenesis, and lymphatic EndMT. While there are limitations to our current research, it sets the stage for further investigations that could lead to new therapeutic approaches for chronic kidney disease and ultimately improve patient outcomes and quality of life.

## Materials and methods

### Animal studies and sample collection

All animal experiments were performed with the approval of the Animal Care Committee at the Hebei University of Chinese Medicine. Seven-week-old male Sprague-Dawley (SD) rats were purchased from Hebei Medical University Animal Center (Hebei, China). The animals were housed at the Hebei University of Chinese Medicine Key Laboratory of Integrative Medicine on a 12-h day/night cycle and were allowed free access to food and water. To investigate the involvement of lymphangiogenesis in the development of contralateral renal fibrosis, male SD rats (*n*= 30) were divided into three groups: the Sham group, the UUO group, and UUO + eplerenone group (each group*n*= 10). In the establishment of UUO model, an incision was made in the midline of the abdomen, and the left proximal ureter was exposed by ligation of the left ureter at the ureteropelvic junction using 3-0 silk. In the eplerenone group, rats subjected to UUO were administered 100 mg·kg^−1^d^−^eplerenone (EPL, Pfizer, USA) for 6 months, which was added to their food, and rats in the other groups were fed regular chow. In the Sham group, the left ureter was only exposed without ligation. The purpose of this study is the obstructive contralateral kidney injury in UUO animal models, so the UUO group will be named UUO-CLK group in this experiment.

Kidney samples were harvested at the end of day 180. After the rats were decapitated under isoflurane anesthesia, the right kidney was removed; sections of the kidney were fixed in 10% formalin and embedded in paraffin for histological evaluation. The remainder of the kidney was snap-frozen in liquid nitrogen and stored at − 80 °C for mRNA and protein analysis.

### Histological examination and immunohistochemical analysis

Kidney sections were histologically evaluated with HE staining. HE staining was performed using standard procedures, and semiquantitative grading was also performed based on a previous method^33^.

For immunohistochemistry, we performed antigen retrieval on kidney sections using a citric acid solution (pH 6.0, 95 °C–100°C) for 20 min. The sections were then incubated with 3% H_2_O_2_for 10 min and blocked with 10% goat serum for 30 min. Primary antibodies against Collagen I (Abcam, Cat#: ab270993), Collagen III (Abcam, Cat#: ab283694), α-SMA (ABclonal, Cat#: A17910), Vimentin (Abcam, Cat#: ab8978), LYVE-1 (Novus Biologicals, Cat#: NB600-1008), VEGF-C (Immunoway, Cat#: YT5297), VEGFR3 (Immunoway, Cat#: YT5878), F4/80 (Invitrogen, Cat#: PA5-21,399), podoplanin (Bioss, Cat#: AD06234578), and Prox-1 (Abcam, Cat#: ab199359) were added and incubated at 4 °C overnight, followed by incubation with the corresponding secondary antibodies for one hour at room temperature. Positive staining signals were visualized by the peroxidase substrate 3,3´-diaminobenzidine (DAB) (ZSGB-BIO, Cat#: 9000), and the sections were subsequently counterstained with diluted hematoxylin.

### Immunofluorescence analysis

Immunofluorescence staining was performed as described previously^23^. Briefly, rat kidneys were fixed with 10% neutral buffered formalin, cryoprotected in 10% sucrose in PBS (6 h at 4 °C), immersed overnight in 20% sucrose in PBS at 4 °C, and frozen in OCT Tissue-Tek compound (SAKURA, Cat#: 4583) before 6 μm-thick cryosections were prepared. Anti-VEGFC (Immunoway, Cat#: YT5297), anti-CD68 (Abcam, Cat#: ab955), anti-LYVE-1 (Novus Biologicals, Cat#: NB600-1008), anti-α-SMA (Abcam, Cat#: ab202509), anti-vimentin (Abcam, Cat#: ab8978) and anti-collagen I (Abcam, Cat#: ab270993) were used to examined the frozen kidney sections. Secondary antibodies conjugated with Alexa 488 (Abcam, Cat#: ab150113, ab150081), Alexa 555 (Abcam, Cat#: ab150078, ab150106) and DyLight 405 (Abcam, Cat#: ab175651) were used to visualize antigen-antibody complexes. Nuclei were stained with 4’,6-diamidino-2-phenylindole (DAPI). Digital images were then obtained by confocal scanning microscopy (CTS SP8, Leica, Germany).

### Western blot analysis

For total protein isolation, tissues and cells were homogenized with protease and phosphatase inhibitors. Nucleoprotein extraction was performed using a kit according to the manufacturer’s instructions (Solarbio, Cat#: R0050). The proteins were subjected to SDS-PAGE and transferred to PVDF membranes. After blocking nonspecific binding with 5% nonfat milk, the membranes were incubated with primary antibodies against VEGFR3 (Immunoway, Cat#: YT5878), LYVE-1 (ABclonal, Cat#:AF4202), podoplanin (Bioss, Cat#: AD06234578), Prox-1 (Abcam, Cat#: ab199359), TNF-α (Servicebio, Cat#: GB11186-100), IL-1β (Affinity, Cat#: AF5103), and NR3C2 (Proteintech, Cat#: 21,854-1-AP) at a 1:500-1:1000 dilution overnight at 4 °C. The next day, the blots were incubated with fluorescein-conjugated secondary antibodies for 1 h at room temperature. Quantification was performed by a Dual-Color Infrared Laser Imaging Scanner (Odyssey, Licor, USA). GAPDH (Proteintech, Cat#:60,004-1-lg), β-actin (Affinity, Cat#: T0022) or β-Tubulin (Affinity, Cat#: T0023) antibodies were used as controls for total proteins, and PCNA (Proteintech, Cat#: 10,205-2-AP) was used as the control for nucleoproteins.

### In vitro cell culture assays

RAW 264.7 cells were purchased from Procell Life Science & Technology Co., Ltd. (Wuhan, China) and cultured in DMEM supplemented with 10% heat-inactivated fetal bovine serum. hLECs were purchased from ScienCell™ Research Laboratories (Carlsbad, CA, USA) and maintained in endothelial cell medium (ECM) (ScienCell, Carlsbad, CA, USA) supplemented with 5% fetal bovine serum, 1% endothelial cell growth supplement, and 1% antibiotic solution. RAW264.7 macrophages were divided into three groups: control group (CON), which was provided normal medium; the aldosterone group (ALD), which was provided with medium containing 10^−7^ mol/L aldosterone (Cayman Chemical, USA); and the eplerenone group (EPL), in which the cells were pretreated with 10^−6^ mol/L eplerenone (Abcam, Cat#: ab141251) for 1 h prior to aldosterone treatment. The proteins were extracted, the corresponding target proteins were examined. hLECs were grouped and treated as described above. In addition, cultured hLECs were treated with 100 ng/ml VEGF-C, and cell growth, wound healing, and tube formation ability were examined in the CON, ALD, and EPL groups.

### Immunocytofluorescence analysis

The dried and sterilized glass slides were placed in a 24-well Petri dish, the cells were spread evenly on the glass slides in the wells. After aldosterone and eplerenone treatment, 4% PFA was added and incubated for 20 min at room temperature. Then, 0.25% Triton X-100 was added and incubated for 15 min for permeabilization, and 10% normal goat serum was added and incubated for 30 min for blocking. Subsequently, the cells were incubated with the primary antibody against NR3C2 (Abcam, Cat#: ab64457) overnight at 4 °C, followed by incubation with the relevant secondary antibody at 37 °C in the dark for one hour for fluorescence staining. DAPI was used for nuclear staining.

### Tube formation assays

A 24-well plate was precoated with ECM gel (Sigma-Aldrich) (200 μl/well). After the Matrigel had polymerized at 37 °C for one hour, hLECs were seeded in each well at a density of 1 × 10^5^cells/200 μL/well. After ten hours of incubation in ECM, tube-like structures were imaged by phase-contrast microscopy (DMi1, Leica) with 10 × objective lenses. Tube length was quantified using ImageJ (US National Institutes of Health, Bethesda, MD, USA).

### Wound healing assays

hLECs were cultured in ECM until they reached confluence. Then, the monolayers were wounded (T0) by creating a scratch across the well with a 200-μl pipette tip. The medium was then replaced with medium containing VEGF-C in the CON, ALD, and EPL groups. The scratches were photographed at 0 h and after 10 h (T0 and T10) using an inverted microscope.

### Flow cytometry

hLECs were treated with aldosterone and analyzed by flow cytometry. The cells were collected and stained with anti-LYVE-1 (Novus Biologicals, Cat#: NB600-1008), after which they were incubated with Alexa 647-labeled goat anti-rabbit IgG (Abcam, Cat#: ab150079) or PE-conjugated anti-VEGFR3 (Biolegend, Cat#: 356,204) secondary antibodies for 1 h. If intracellular staining was needed, a fixation and permeabilization procedure was used (eBioscience™ Foxp3, Invitrogen, Cat#: 00-5523-00). Then, the cells were stained with Alexa Fluor® 647-conjugated anti-α-SMA (Abcam, Cat#: ab202296), APC-conjugated anti-vimentin (Invitrogen, Cat#: MA5-28,801) and FITC-conjugated anti-Ki67 (Invitrogen, Cat: 11-5698-82) for 1 h in the dark. The cells were then analyzed on a BD Accuri C6 flow cytometer.

### Ethical approval

All experiments were carried out in accordance with recommendations for the Care and Use of Laboratory Animals in the National Institutes of Health Guidelines. Animal care followed the criteria of the Ethics Committee of Hebei University of Chinese Medicine. The authors complied with the ARRIVE guidelines.

### Statistical analysis

Statistical analysis was performed using IBM SPSS Statistics, version 23.0 (https://www.ibm.com/products/spss-statistics) (IBM, Armonk, NY, USA). The results are presented as the means ± standard deviation. Differences between groups were compared by one-way analysis of variance (ANOVA) followed by the least significant difference (LSD) test. For all the statistical tests, a two-tailed*P*value < 0.05 was considered to indicate statistical significance.

### Supplementary Information

Supplementary Figures.

## Data Availability

The original contributions presented in the study are available from the corresponding author, QX, upon reasonable request.

## References

[1] Carney EF (2020). The impact of chronic kidney disease on global health. Nat. Rev. Nephrol..

[2] Jha V (2013). Chronic kidney disease: Global dimension and perspectives. The Lancet.

[3] Lv JC, Zhang LX (2019). Prevalence and disease burden of chronic kidney disease. Adv. Exp. Med. Biol..

[4] Pei G (2019). Lymphangiogenesis in kidney and lymph node mediates renal inflammation and fibrosis. Sci. Adv..

[5] Ma X (2019). Eplerenone ameliorates cell pyroptosis in contralateral kidneys of rats with unilateral ureteral obstruction. Nephron.

[6] Wang C-H (2017). The inhibitory effect of eplerenone on cell proliferation in the contralateral kidneys of rats with unilateral ureteral obstruction. Nephron.

[7] Xiong Y (2021). Eplerenone attenuates fibrosis in the contralateral kidney of UUO rats by preventing macrophage-to-myofibroblast transition. Front. Pharmacol..

[8] Guo YC (2017). macrophages regulate unilateral ureteral obstruction-induced renal lymphangiogenesis through C-C motif chemokine receptor 2-dependent phosphatidylinositol 3-Kinase-AKT-mechanistic target of rapamycin signaling and hypoxia-inducible factor-1alpha/vascular endothelial growth factor-C expression. Am. J. Pathol..

[9] Meng XM (2016). Inflammatory macrophages can transdifferentiate into myofibroblasts during renal fibrosis. Cell Death Dis..

[10] Vernon MA, Mylonas KJ, Hughes J (2010). Macrophages and renal fibrosis. Seminars Nephrol..

[11] Chrissobolis S (2017). Vascular consequences of aldosterone excess and mineralocorticoid receptor antagonism. Curr. Hypertens. Rev..

[12] Pacurari M, Kafoury R, Tchounwou PB, Ndebele K (2014). The renin-angiotensin-aldosterone system in vascular inflammation and remodeling. Int. J. Inflamm..

[13] Seeger H, Bonani M, Segerer S (2012). The role of lymphatics in renal inflammation. Nephrol. Dial. Transp..

[14] Tammela T, Alitalo K (2010). Lymphangiogenesis: Molecular mechanisms and future promise. Cell.

[15] Vaahtomeri K, Karaman S, Mäkinen T, Alitalo K (2017). Lymphangiogenesis guidance by paracrine and pericellular factors. Genes Dev..

[16] Kinashi H, Ito Y, Sun T, Katsuno T, Takei Y (2018). Roles of the TGF-beta (–) VEGF-C pathway in fibrosis-related lymphangiogenesis. Int. J. Mol. Sci..

[17] Lee AS (2013). Vascular endothelial growth factor-C and -D are involved in lymphangiogenesis in mouse unilateral ureteral obstruction. Kidney Int..

[18] Donnan MD (2021). The lymphatics in kidney health and disease. Nat. Rev. Nephrol..

[19] Wu J (2020). Lymphatic vessels enhancing adaptive immunity deteriorates renal inflammation and renal fibrosis. Kidney Dis. Basel..

[20] Zimmer JK (2010). Lymphangiogenesis is upregulated in kidneys of patients with multiple myeloma. Anat. Rec. Hoboken.

[21] Jafree DJ (2020). Beyond a passive conduit: Implications of lymphatic biology for kidney diseases. J. Am. Soc. Nephrol..

[22] Wei H (2022). CD137L-macrophage induce lymphatic endothelial cells autophagy to promote lymphangiogenesis in renal fibrosis. Int. J. Biol. Sci..

[23] Qiang P (2022). Esaxerenone inhibits the macrophage-to-myofibroblast transition through mineralocorticoid receptor/TGF-β1 pathway in mice induced with aldosterone. Front. Immunol..

[24] Suzuki Y (2012). Transforming growth factor-b induces vascular endothelial growth factor-C expression leading to lymphangiogenesis in rat unilateral ureteral obstruction. Kidney Int..

[25] Long DA (2012). Restoring the renal microvasculature to treat chronic kidney disease. Nat. Rev. Nephrol..

[26] Haase VH (2013). Mechanisms of hypoxia responses in renal tissue. J. Am. Soc. Nephrol..

[27] Yazdani S (2012). Proteinuria triggers renal lymphangiogenesis prior to the development of interstitial fibrosis. PLoS One.

[28] Chen G (2022). Eplerenone inhibits UUO-induced lymphangiogenesis and cardiac fibrosis by attenuating inflammatory injury. Int. Immunopharmacol..

[29] Rosa I (2023). Lymphatic endothelial-to-myofibroblast transition: A potential new mechanism underlying skin fibrosis in systemic sclerosis. Cells.

[30] Yoshimatsu Y (2020). TGF-beta and TNF-alpha cooperatively induce mesenchymal transition of lymphatic endothelial cells via activation of Activin signals. PloS One.

[31] Liu J (2022). Lymphangiogenesis and lymphatic barrier dysfunction in renal fibrosis. Int. J. Mol. Sci..

[32] Georgianos PI, Agarwal R (2021). Mineralocorticoid receptor antagonism in chronic kidney disease. Kidney Int. Rep..

[33] Xiong Y (2021). Eplerenone attenuates fibrosis in the contralateral kidney of UUO rats by preventing macrophage-to-myofibroblast transition. Front. Pharmacol..

