# Supplementary material for: Eplerenone reduces lymphangiogenesis in the contralateral kidneys of UUO rats
Source: Sci Rep. 2024 May 1;14:9976. doi: 10.1038/s41598-024-60636-z (PMC11063175; doi:10.1038/s41598-024-60636-z)
Supplement: Supplementary file 1 — Supplementary Figures. [file 41598_2024_60636_MOESM1_ESM.pptx]

## Slide 1
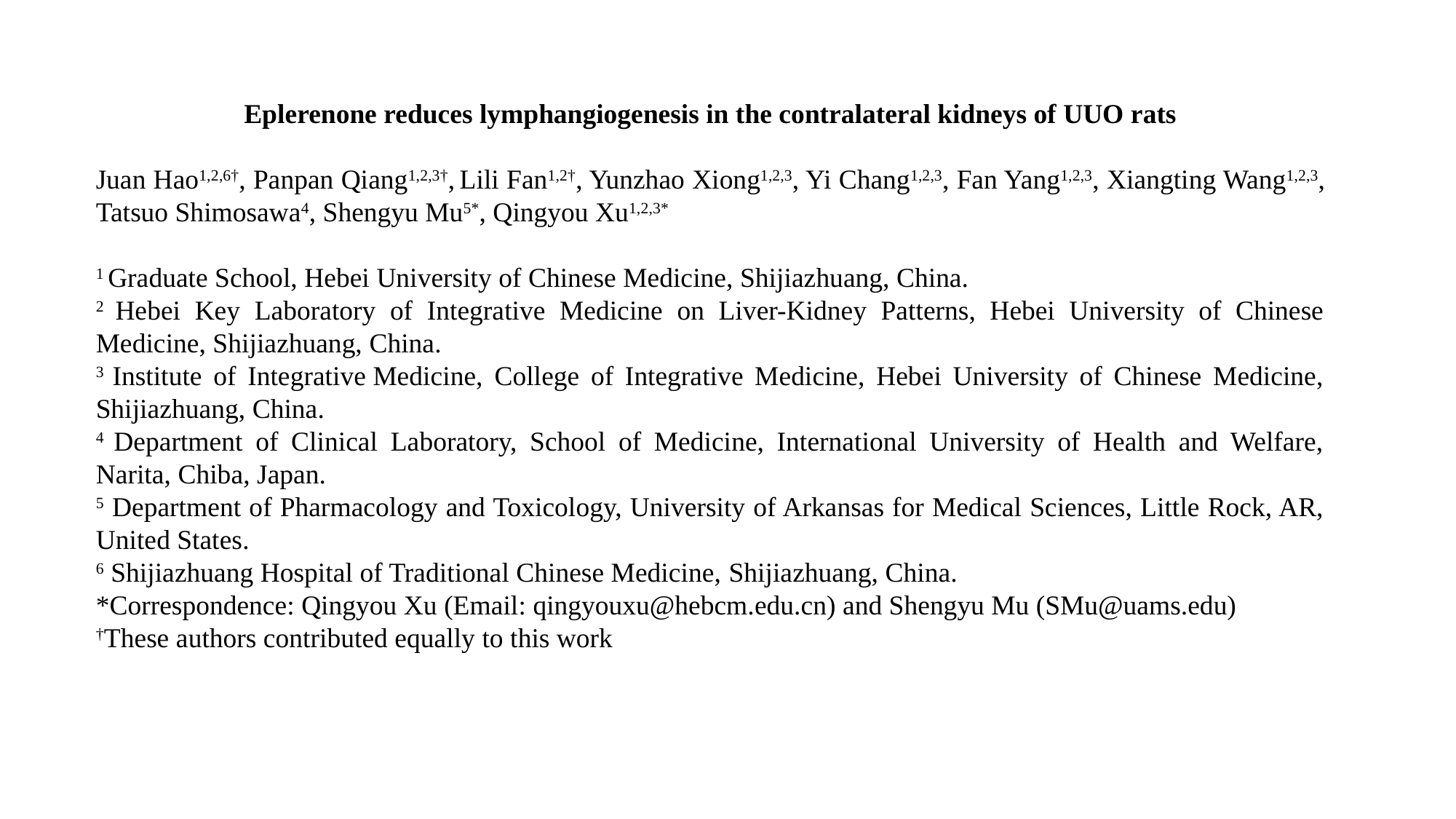

Eplerenone reduces lymphangiogenesis in the contralateral kidneys of UUO rats
Juan Hao1,2,6†, Panpan Qiang1,2,3†, Lili Fan1,2†, Yunzhao Xiong1,2,3, Yi Chang1,2,3, Fan Yang1,2,3, Xiangting Wang1,2,3, Tatsuo Shimosawa4, Shengyu Mu5*, Qingyou Xu1,2,3*
1 Graduate School, Hebei University of Chinese Medicine, Shijiazhuang, China.
2 Hebei Key Laboratory of Integrative Medicine on Liver-Kidney Patterns, Hebei University of Chinese Medicine, Shijiazhuang, China.
3 Institute of Integrative Medicine, College of Integrative Medicine, Hebei University of Chinese Medicine, Shijiazhuang, China.
4 Department of Clinical Laboratory, School of Medicine, International University of Health and Welfare, Narita, Chiba, Japan.
5 Department of Pharmacology and Toxicology, University of Arkansas for Medical Sciences, Little Rock, AR, United States.
6 Shijiazhuang Hospital of Traditional Chinese Medicine, Shijiazhuang, China.
*Correspondence: Qingyou Xu (Email: qingyouxu@hebcm.edu.cn) and Shengyu Mu (SMu@uams.edu)
†These authors contributed equally to this work

## Slide 2
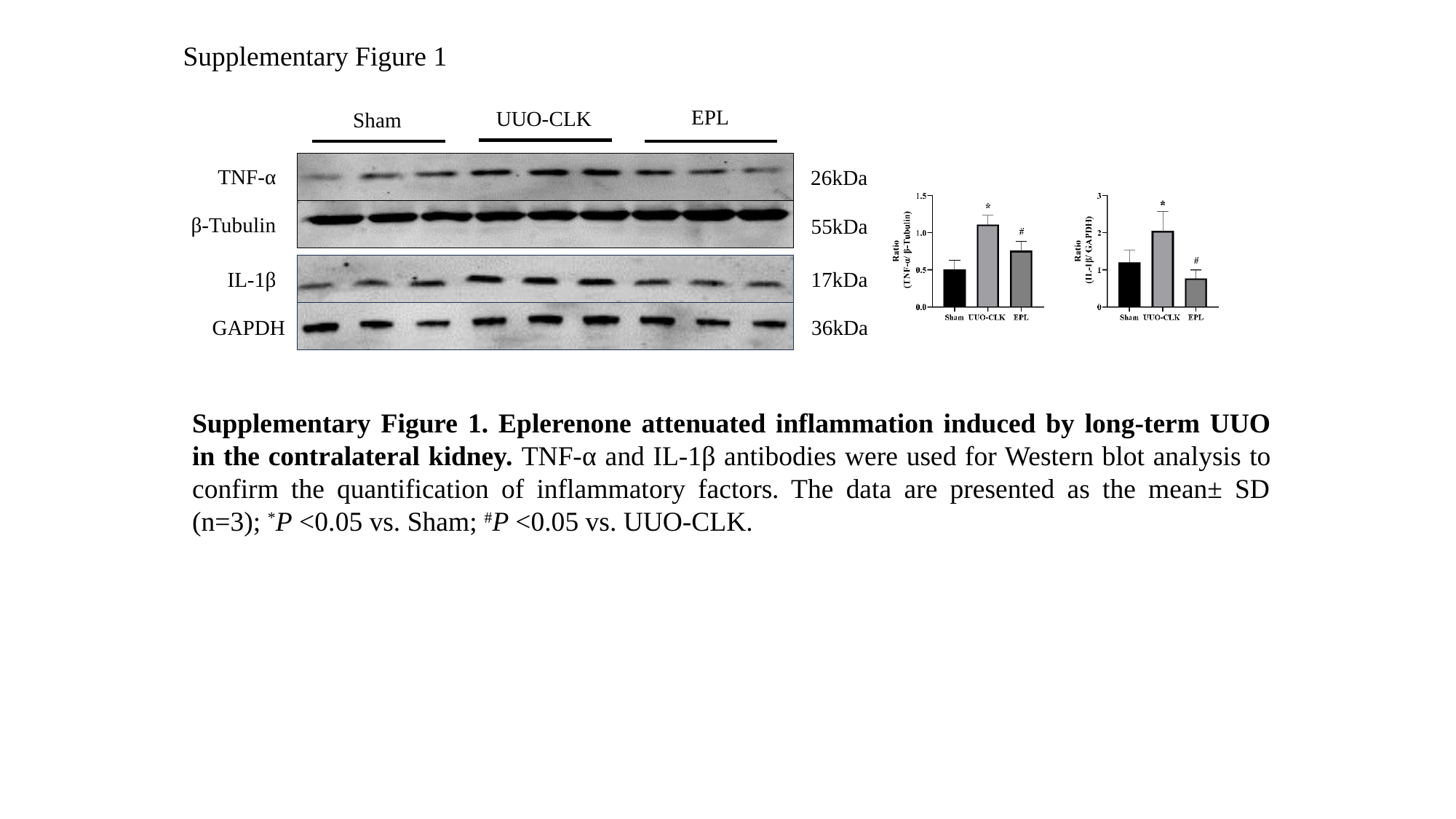

Supplementary Figure 1
EPL
UUO-CLK
Sham
TNF-α
26kDa
55kDa
β-Tubulin
17kDa
36kDa
IL-1β
GAPDH
Supplementary Figure 1. Eplerenone attenuated inflammation induced by long-term UUO in the contralateral kidney. TNF-α and IL-1β antibodies were used for Western blot analysis to confirm the quantification of inflammatory factors. The data are presented as the mean± SD (n=3); *P <0.05 vs. Sham; #P <0.05 vs. UUO-CLK.

## Slide 3
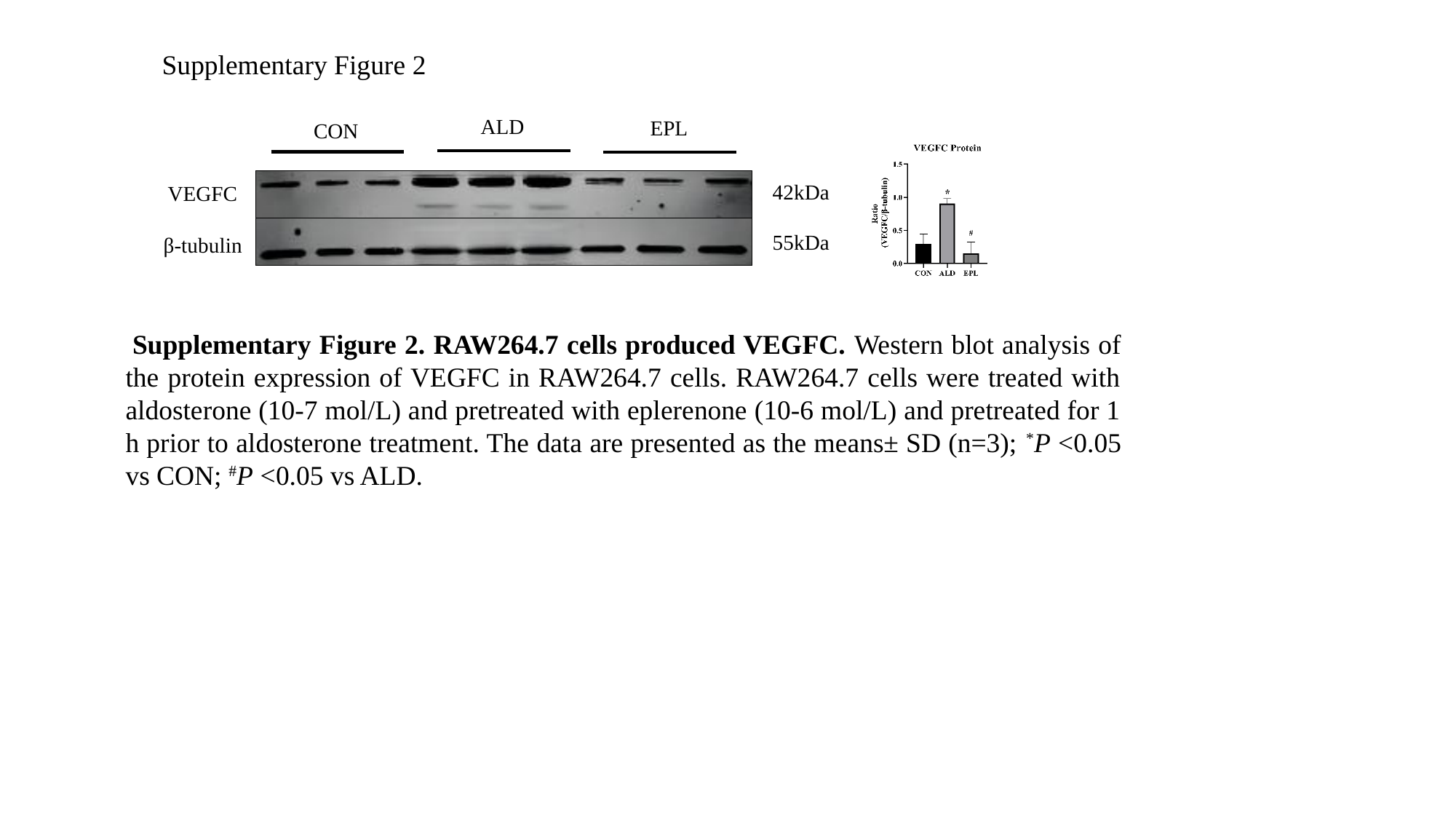

Supplementary Figure 2
ALD
EPL
CON
42kDa
VEGFC
55kDa
β-tubulin
 Supplementary Figure 2. RAW264.7 cells produced VEGFC. Western blot analysis of the protein expression of VEGFC in RAW264.7 cells. RAW264.7 cells were treated with aldosterone (10-7 mol/L) and pretreated with eplerenone (10-6 mol/L) and pretreated for 1 h prior to aldosterone treatment. The data are presented as the means± SD (n=3); *P <0.05 vs CON; #P <0.05 vs ALD.

## Slide 4
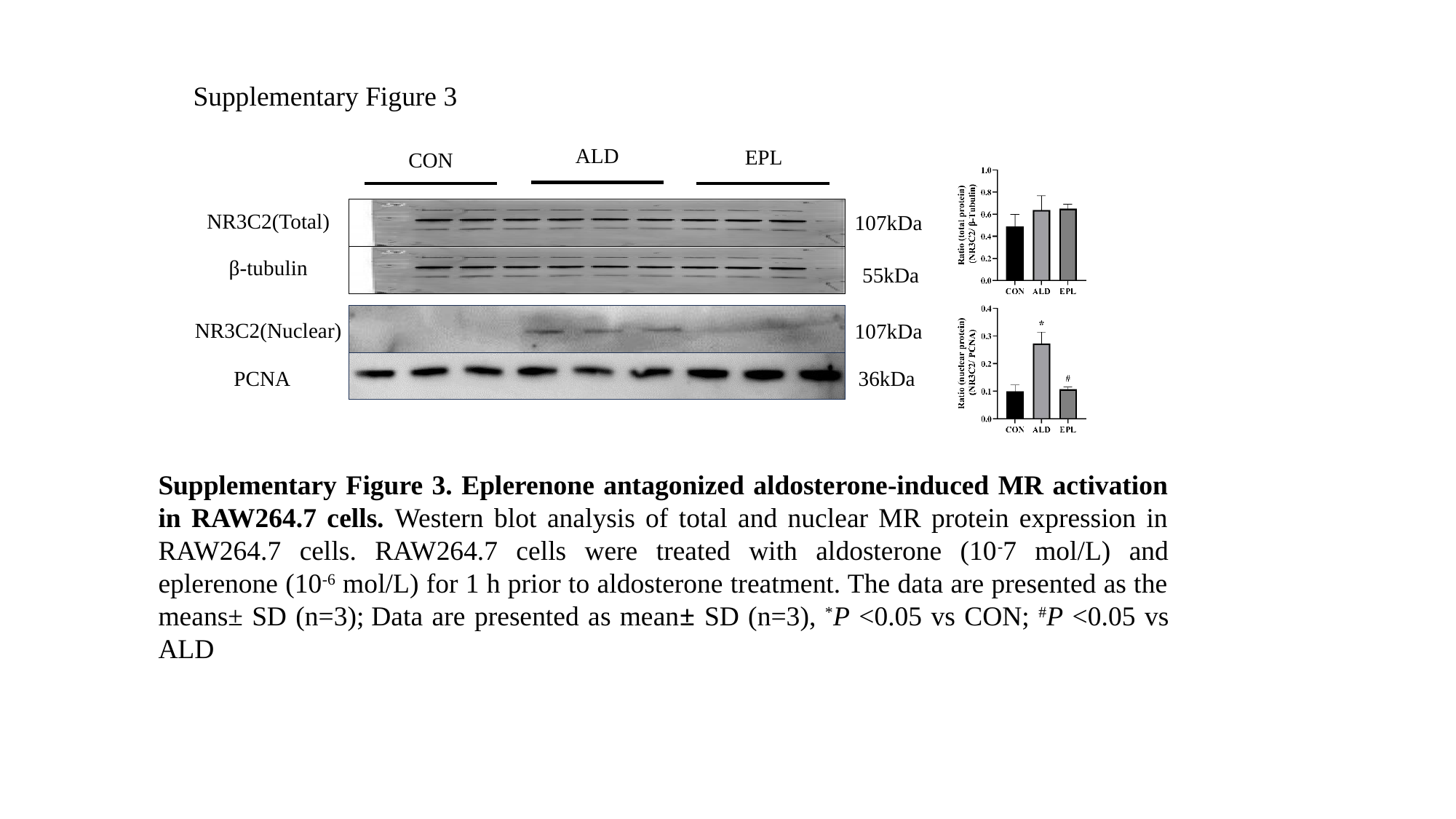

Supplementary Figure 3
ALD
EPL
CON
NR3C2(Total)
107kDa
β-tubulin
55kDa
NR3C2(Nuclear)
107kDa
PCNA
36kDa
Supplementary Figure 3. Eplerenone antagonized aldosterone-induced MR activation in RAW264.7 cells. Western blot analysis of total and nuclear MR protein expression in RAW264.7 cells. RAW264.7 cells were treated with aldosterone (10-7 mol/L) and eplerenone (10-6 mol/L) for 1 h prior to aldosterone treatment. The data are presented as the means± SD (n=3); Data are presented as mean± SD (n=3), *P <0.05 vs CON; #P <0.05 vs ALD

## Slide 5
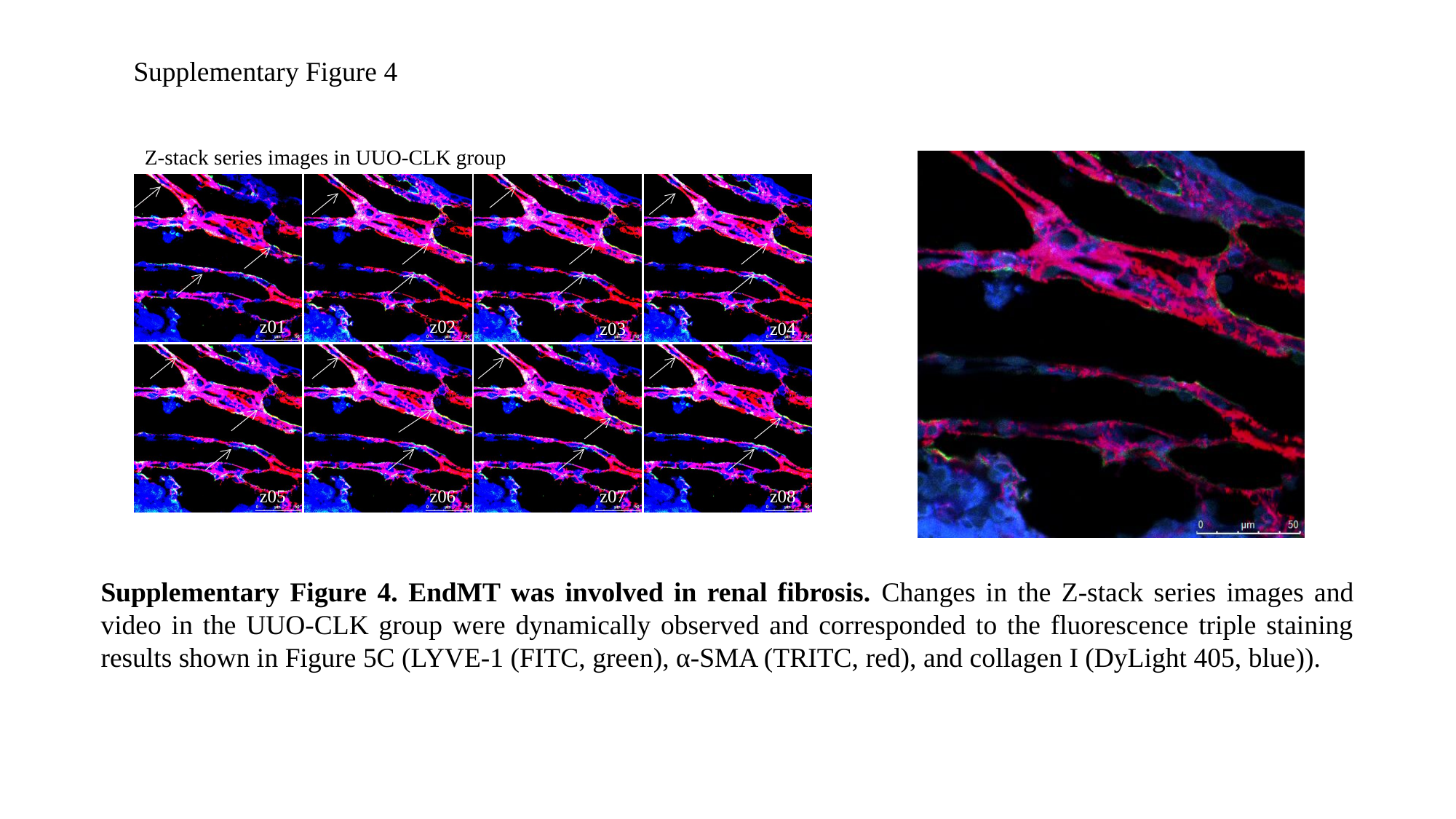

Supplementary Figure 4
Z-stack series images in UUO-CLK group
z01
z02
z03
z04
z05
z06
z07
z08
Supplementary Figure 4. EndMT was involved in renal fibrosis. Changes in the Z-stack series images and video in the UUO-CLK group were dynamically observed and corresponded to the fluorescence triple staining results shown in Figure 5C (LYVE-1 (FITC, green), α-SMA (TRITC, red), and collagen I (DyLight 405, blue)).
